# Visual, Verbal and Everyday Memory 2 Years After Bariatric Surgery: Poorer Memory Performance at 1-Year Follow-Up

**DOI:** 10.3389/fpsyg.2020.607834

**Published:** 2021-01-08

**Authors:** Gro Walø-Syversen, Ingela L. Kvalem, Jon Kristinsson, Inger L. Eribe, Øyvind Rø, Cathrine Brunborg, Camilla Lindvall Dahlgren

**Affiliations:** ^1^Regional Department for Eating Disorders, Oslo University Hospital, Oslo, Norway; ^2^Department of Psychology, University of Oslo, Oslo, Norway; ^3^Centre for Morbid Obesity and Bariatric Surgery, Oslo University Hospital, Oslo, Norway; ^4^Institute of Clinical Medicine, Faculty of Medicine, University of Oslo, Oslo, Norway; ^5^Oslo Centre for Biostatistics and Epidemiology, Research Support Services, Oslo University Hospital, Oslo, Norway; ^6^Department of Psychology, Bjørknes University College, Oslo, Norway

**Keywords:** visual memory, verbal memory, everyday memory, bariatric surgery, postoperative, weight loss

## Abstract

Severe obesity has been associated with reduced performance on tests of verbal memory in bariatric surgery candidates. There is also some evidence that bariatric surgery leads to improved verbal memory, yet these findings need further elucidation. Little is known regarding postoperative memory changes in the visual domain and how patients subjectively experience their everyday memory after surgery. The aim of the current study was to repeat and extend prior findings on postoperative memory by investigating visual, verbal, and self-reported everyday memory following surgery, and to examine whether weight loss and somatic comorbidity predict memory performance. The study was a prospective, observational study in which participants (*n* = 48) underwent cognitive testing at baseline, 1 and 2 years after bariatric surgery. Repeated measures analyses of variance revealed significantly poorer visual and verbal memory performance at the 1-year follow-up, with performance subsequently returning to baseline levels after 2 years. Verbal learning and self-reported everyday memory did not show significant postoperative changes. Memory performance at 1 year was not significantly predicted by weight loss, changes in C-reactive protein levels or postoperative somatic comorbidity (Type 2 diabetes, sleep apnea, and hypertension). The study demonstrated poorer visual and verbal memory performance at 1-year follow-up that returned to baseline levels after 2 years. These findings are in contrast to most previous studies and require further replication, however, the results indicate that postoperative memory improvements following bariatric surgery are not universal. Findings suggest that treatment providers should also be aware of patients potentially having poorer memory at 1 year following surgery.

## Introduction

Over the past decade, increasing evidence suggests that severe obesity has adverse effects on cognitive functioning, including episodic memory (Nguyen et al., 2014; Prickett et al., 2015; Spitznagel et al., 2015; Dye et al., 2017; Loprinzi and Frith, 2018; Farruggia and Small, 2019). Epidemiological studies indicate that midlife obesity increases the risk for late-life dementia development (Anstey et al., 2011; Xu et al., 2011; Pedditizi et al., 2016; Singh-Manoux et al., 2018). Obesity has also been linked to metabolic and structural changes in brain areas that support memory processes (Spitznagel et al., 2015; Nota et al., 2020), such as the hippocampus (Raji et al., 2010) and prefrontal cortex (Volkow et al., 2009; Kurth et al., 2013; Yau et al., 2014). Moreover, neuropsychological studies have demonstrated associations between severe obesity and reduced performance on cognitive tests of visual memory (e.g., remembering visual patterns) (Gunstad et al., 2010; Sargénius et al., 2017), prospective memory (e.g., remembering activities to be performed) (Gunstad et al., 2010), “what-where-when” memory (e.g., memory for complex events) (Cheke, 2016; Cheke et al., 2017) and verbal memory (e.g., learning and remembering verbally presented material) (Gunstad et al., 2006; Hartanto and Yong, 2018). Further, studies have shown that a significant proportion of bariatric surgery patients present with impaired episodic memory performance, as indicated by word-list-learning performance, prior to surgery (Gunstad et al., 2011; Miller et al., 2013; Alosco et al., 2014a,b). Episodic memory is central to adequate functioning in everyday life (Lezak, 2012) and is also one of the key processes involved in the regulation of eating behaviors (Higgs and Spetter, 2018). As outlined in a recent review (Higgs and Spetter, 2018), experimental meal memory paradigms have shown that manipulating memory for a recent meal affect later food intake (Higgs et al., 2008). In addition, both clinical data (e.g., overeating in amnesic patients) (Rozin et al., 1998) and non-clinical studies (Attuquayefio et al., 2016; Martin et al., 2018) point to the role of general memory in appetite dysregulation, uncontrolled eating and weight gain. These findings have clinical implications for bariatric surgery patients, as memory difficulties may lead to challenges in adhering to specific pre- and postoperative dietary advice and more general health regimens.

Notably, bariatric surgery is associated with improvements in postoperative verbal memory performance (Saindane et al., 2019). Literature has indicated that both behavioral and surgical weight loss interventions are related to improvements in several cognitive domains (e.g., attention, executive function, and memory), independent of baseline BMI (Veronese et al., 2017), and weight loss is assumed to impede obesity-related cognitive decline (Siervo et al., 2011; Handley et al., 2016; Thiara et al., 2017; Veronese et al., 2017). Bariatric surgery, which is the most effective intervention for substantial and sustained weight loss (Chang et al., 2014), has been posited as a particularly important contributor in attenuating the potentially negative consequences of severe obesity on cognitive function (Stanek and Gunstad, 2013; Haley et al., 2015; Spitznagel et al., 2015; Saindane et al., 2019; Nota et al., 2020). Several publications from the Longitudinal Assessment of Bariatric Surgery (LABS) study have demonstrated reduced performance on cognitive tests in bariatric surgery candidates (Spitznagel et al., 2015), with 12–23% of these patients showing clinically significant verbal memory impairment prior to surgery (Gunstad et al., 2011). Moreover, some of these studies have shown that verbal memory performance tends to moderately improve at 12 weeks (Gunstad et al., 2011), 1 year (Miller et al., 2013), 2 years (Alosco et al., 2014b), and 3 years after surgery (Alosco et al., 2014a). However, in these specific studies, memory improvements were not strongly associated with the amount of weight loss. Prior reviews (Spitznagel et al., 2015; Handley et al., 2016; Nota et al., 2020) have emphasized that the weight loss-related resolution of somatic comorbidities might play a more important role, including Type 2 diabetes (T2D) (Cheke et al., 2017), hypertension (Walker et al., 2017), sleep apnea (Olaithe et al., 2018) and metabolic inflammation (often measured via C-reactive protein (CRP) levels) (Allison and Ditor, 2014).

Research on postoperative changes in memory performance might profit from additional investigations, as the evidence for postoperative memory improvements remains incomplete.

First, prior studies have mainly focused on verbal memory, limiting our understanding of postoperative change in visual memory. In order to comprehensively investigate postoperative memory, inclusion of non-verbal memory tests, which are less affected by language ability and cultural context (Sahakian and Owen, 1992), is important. In addition, prior studies have associated severe obesity with reduced performance on visual memory tests (Prickett et al., 2015), but few studies on postoperative visual memory performance are found in the literature (Pearce et al., 2019; Saindane et al., 2019). Second, knowledge of patients’ subjective experience of postoperative memory difficulties is scarce and we know little about self-reported memory in this patient group. Only one prior study (Garcia et al., 2013) has investigated self-reported cognitive problems in bariatric surgery candidates using three generic questions, limiting further comparison to relevant studies from other clinical populations. Last, as memory functioning following surgery have clinical importance due to its likely role in postoperative treatment adherence, it is important to repeat and extend findings from prior studies and to test whether findings of postoperative verbal memory improvements are generalizable to populations from other countries (Handley et al., 2016).

The aim of this study was to investigate postoperative verbal, visual, and everyday memory using a combination of performance-based tests and self-report, in a sample of bariatric surgery patients at 1 and 2 years after bariatric surgery. In addition to investigating postoperative memory, we examined whether weight loss and postoperative comorbidity predicted memory performance after surgery.

## Materials and Methods

### Study Design, Procedures, and Participants

**FIGURE 1:**
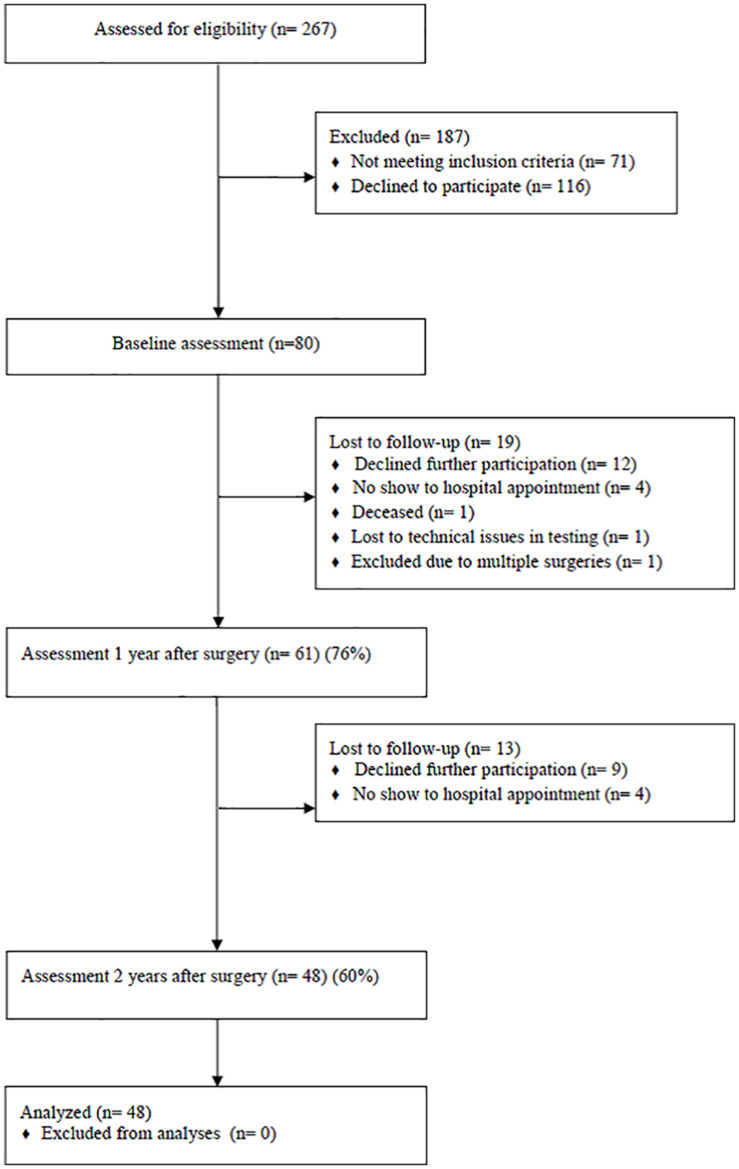
Flow chart of recruitment and data collection.

The study is based on a subset of data from a prospective, observational study investigating cognitive function (Oslo Bariatric Surgery Study Cognitive, OBSSC) in bariatric surgery patients over a 2-year follow-up period (Walø-Syversen et al., 2019). The study was approved by the Regional Committee for Medical and Health Research Ethics of South-Eastern Norway and the Privacy Ombudsman for Research at Oslo University Hospital. Informed consent was obtained for all study participants. The study recruitment (June 2016 to May 2017) and data collection process is presented in Figure 1. All patients (*n* = 267) scheduled for surgery during the recruitment period were asked to participate in the study. Exclusion criteria were the presence of a neurological disorder, moderate/severe head injury, past/present history of severe psychiatric illness, past/current alcohol or drug abuse/dependence, history of a learning disorder, developmental disability and impaired sensorimotor function. The neuropsychological testing was scheduled 30 days (±5 days) before the operation, 1 year (±2 weeks), and 2 years (±2 weeks) after surgery. Self-report questionnaires were completed at home prior to the testing. Follow-up appointments were scheduled to occur on the same day as clinical visits to the hospital. There were no significant differences in terms of age or male to female ratio between participants that agreed to participate in the study at baseline (*n* = 80) vs. patients scheduled for surgery in the recruitment period who did not participate (*n* = 187). Due to the loss of participants at 1- and 2-year follow-up, the final sample size was 48 (Figure 1). Analyses of all study baseline variables; age [*t*(78) = 1.29, *p* = 0.20], body mass index (BMI) [*t*(78) = −0.98, *p* = 0.32], verbal learning [*t*(78) = 1.46, *p* = 0.14), recognition (t (77) = 0.54, *p* = 0.58], short delay free recall [*t*(77) = 0.69, *p* = 0.49], long delay free recall [*t*(77) = 0.87, *p* = 0.38], visual memory [*t*(78) = −0.54, *p* = 0.59], and everyday memory [*t*(78) = 0.36, *p* = 0.71], showed no significant differences between the final sample of participants (*n* = 48) and participants that were lost to follow-up (*n* = 32).

### Measures

The *Paired Associate Learning (PAL)* from the Cambridge Neuropsychological Automated Battery (CANTAB) (CANTAB, 2019) is a computerized test of visual paired associate learning and memory. The test is widely used in studies on non-verbal episodic memory across several research disciplines (e.g., psychiatry, neurology, and clinical neuropsychology) (Barnett et al., 2016; Karlsen et al., 2020). The test has numerous matched forms of test stimuli to reduce the risk of practice effects across repeated assessments (Barnett et al., 2016; Cacciamani et al., 2018). Participants are presented for boxes on the screen that open in random order revealing visual patterns. The participants are instructed to memorize in which box each specific pattern was located. Next, the patterns are presented one at a time in the middle of the screen and the participants must select and touch the box in which the pattern was originally located. The number of boxes increases across trials. Performance was measured by adjusted total errors (the number of times the participant touched the incorrect box) converted to normed standard scores derived from the CANTAB normative database, which use z- scores (*M* = 0, *SD* = 1). A higher score indicates better performance.

The *California Verbal Memory Test II (CVLT-II)* (Delis et al., 2000; Lundervold and Sundet, 2004) is a widely used neuropsychological test of verbal learning and memory (Woods et al., 2006), and is one of the commonly used verbal memory tests in prior studies of obesity (Prickett et al., 2015). The test is a list-learning task, yielding a multitude of outcome measures, including recall and recognition of two 16-word lists across immediate and delayed trials. To mitigate practice effects (Benedict, 2005; Elman et al., 2018), which may occur at long test intervals (Alioto et al., 2017), participants were presented with the standard form at baseline, the alternate form (different nouns) at 1-year follow-up, and the standard form at 2-year follow-up. We used total learning (number correct, trials 1–5) (verbal learning), short delay free recall (number correct) (SD free recall), long delay free recall (number correct) (LD free recall) and recognition (total hits) (recognition) as outcome measures. The CVLT-II computerized scoring program was used to obtain normed standard scores for all selected measures. Hence, the measures are reported as z-scores (*M* = 0, *SD* = 1), except for the total learning trial which uses T-score (*M* = 50, *SD* = 10). Higher scores indicate better performance.

The *Everyday Memory Questionnaire Revised* (EMQ-R) (Royle and Lincoln, 2008) is a 13-item self-report questionnaire that assesses everyday memory failures. The EMQ-R was developed from the 28–item Everyday Memory Questionnaire by Sunderland (Sunderland et al., 1983, 1984), and has been used in both clinical and non-clinical samples. The EMQ-R has strong internal reliability and a two-factor structure (labeled Retrieval/Forgetting and Attention/Attentional tracking) has been identified (Royle and Lincoln, 2008; Evans et al., 2020). Participants are asked to assess the rate of memory-related behaviors (i.e., forgetting important details of what you did or what happened to you the day before) over the last month. Frequency of forgetting is rated on the following scale: 0 = once or less in the past month, 1 = more than once a month but less than once a week, 2 = about once a week, 3 = more than once a week but less than once a day, 4 = once or more in a day. Items are totaled (range 0–52), and a higher sum score indicates greater *presence* of everyday memory problems. An average score of memory *functioning* (0–4) was also calculated. Due to uncertainty related to factor structure and reliability in our sample, a factor analysis and internal reliability analysis were performed on baseline sample data (*n* = 80). The analysis revealed a two-factor solution that accounted for 60.27% of the variance (see Supplementary Material). Cronbach‘s alpha for the total scale was 0.91 at baseline. The total scale was used as the outcome measure (sum score).

#### Comorbidity and Weight

Hypertension was defined as having a systolic blood pressure of ≥140 mm Hg or diastolic blood pressure of ≥90 mm Hg during testing or self-reported use of antihypertensive medication. Presence of sleep apnea and Type 2 diabetes (T2D) was determined based upon clinical diagnoses from participants’ medical records. CRP-levels were determined from blood samples taken at the hospital during routine clinical appointments. Participants’ weight was measured on a platform scale SECA 635, III. They were measured without shoes and wearing light clothes.

### Statistical Analyses

Descriptive statistics were used to characterize the sample and to investigate rates of comorbid disease before and after surgery. In order to facilitate direct comparison to findings from prior studies (Gunstad et al., 2011; Miller et al., 2013; Alosco et al., 2014b), pre- and postoperative rates of impairment in three verbal memory measures (SD free recall, LD free recall, and recognition) were examined. Similar to these reports, impairment was defined as test performance ≥1.5 standard deviation (SD) below the normative mean (Tanner-Eggen et al., 2015). McNemar’s test compared rates of comorbid disease and clinically significant verbal memory impairment from baseline to 1 year after surgery. Histograms and Quantile-Quantile Plots were used to check all continuous variables for normality. Repeated measures (RM) ANOVA with *post-hoc* Bonferroni corrected pairwise comparisons were conducted to identify significant changes in weight and outcome measures (visual memory, short delay (SD) free recall, long delay (LD) free recall, recognition, verbal learning and EMQ-R) over time (baseline, 1-year follow-up and 2-year follow-up). The sphericity assumption was violated for all outcome measures, hence Greenhouse-Geisser corrected estimates are reported (Field, 2017). PAL variables were log transformed due to skewness (PAL at 1-year follow-up), but log transformed results were identical to results with original values, hence we report and use original values. There were missing data for the CVLT-II variables (seven values) and the EMQ-R (three individuals without full questionnaire). To avoid data loss we replaced missing values using expectancy maximization (Dong and Peng, 2013). Partial eta squared was used as effect size measure (0.01 = small, 0.06 = medium, and 0.14 = large) (Hahs-Vaughn and Lomax, 2020). Multiple regression (MR) analyses were used to evaluate predictors of postoperative memory change. Predetermined independent variables (IV) were change in absolute weight (weight at baseline testing—follow up weight/weight at baseline testing), change in CRP-levels and the postoperative presence of comorbid disease. All IVs with a *p*-level of < 0.15 in preliminary simple linear regressions were to be included in the final MR models. A significance level of *p* ≤ 0.05 was used. All statistical analyses were performed using SPSS standard version 25. Sample size considerations, which included statistical power analyses conducted in G^∗^power (Faul et al., 2007) and based on prior studies (Miller et al., 2013; Spitznagel et al., 2014), estimated that a sample size of 34 was sufficient for detecting large effects in a multiple linear regression analyses with 10 variables, given a Type 1 error rate of 5% and power of 80%. For the RM ANOVA no *a priori* power analysis was performed. A sensitivity analyses (Faul et al., 2007) estimated that a RM ANOVA with 48 participants and 3 repeated measurements would be sensitive to effects of Cohen’s *f*^2^ = 0.32 with 80% power (Type 1 error rate of 5%), which indicates the study was able to reliably detect effect sizes larger than η^2^*_*p*_* = 0.09 (i.e., medium effect size).

## Results

### Sample Characteristics

Sample characteristics are presented in Table 1. Participants (*n* = 48) were 73% female, mean age was 42.35 (*SD* = 10.47) years, mean years of education was 13.33 (*SD* = 2.27). The type of bariatric surgery included gastric sleeve (21%), Roux-en-Y gastric bypass (RYGB) (54%), and one anastomosis gastric bypass (25%). Percentage total weight loss (%TWL) (weight at baseline testing—follow up weight/weight at baseline testing) x 100%) was 31.58% (7.83) kg at 1 year and 31.76% (8.38) kg at 2 years after surgery.

**TABLE 1:** Sample characteristics and rates of comorbid disease and memory impairment (*n* = 48).

	Baseline	1-year follow-up	2-year follow-up	*p*
Age, mean (SD)	42.35 (10.47)			
Female (%)	73%			
Years of education (mean, SD)	13.33 (2.27)			
BMI (mean, SD)	43.20 (4.56)	29.68 (5.42)	29.63 (5.74)	0.001
Weight (mean, SD)	122.66 (17.93)	84.50 (18.75)	84.37 (19.53)	0.001
Weight loss (mean, SD)		38.15 (8.72)	38.28 (9.00)	0.001
Percentage weight loss (mean, SD)		31.58 (7.83)	31.76 (8.38)	0.001
Sleep apnea (%)	36%	6%	6%	0.001
Type 2 diabetes (%)	23%	4%	4%	0.004
Hypertension (%)	40%	8%	18%	0.001
CRP (median, IQR)	5.55 (3–10)	0.75 (0.6–2)	0.60 (0.6–1.4)	0.001
SD free recall impairment (%)	12%	20%	0%	n.s.
LD free recall impairment (%)	17%	19%	6%	n.s.
Recognition (hits) impairment (%)	8%	19%	4%	n.s.

**SD, standard deviation; CRP, C-reactive protein; SD, short delay; LD, long delay; n.s, not significant. Repeated measures ANOVA compared differences across time for BMI, weight, weight loss, percentage weight loss, and CRP. Level of significance is reported for the main effect (F-test). McNemar’s tests compared the rates of memory impairment and rates of comorbid disease from before surgery to the 1-year follow-up. Presence of verbal memory impairment was defined as test scores 1.5 SD below the normative mean.**

### Comorbid Disease at Baseline and 1-Year Follow-Up

A moderate to large proportion of the participants showed the presence of comorbid disease (T2D, sleep apnea and hypertension) at baseline. The median group CRP-level at baseline was at 5.55, indicating an overall presence of low-grade inflammation. The rate of comorbid disease and CRP-levels significantly decreased at 1-year follow-up (Table 1).

### Preoperative and Postoperative Rates of Impaired Verbal Memory Performance

As seen in Table 1, the rates of impairment (defined as test performance 1.5 SD below the normative mean) in SD free recall, LD free recall and recognition ranged from 8 to 17% at baseline. The rates of verbal memory impairment were unchanged for all three measures from baseline to 1 year follow-up, however, rates were lower at 2-year follow-up (Table 1).

### Postoperative Change in Mean Memory Performance

**FIGURE 2:**
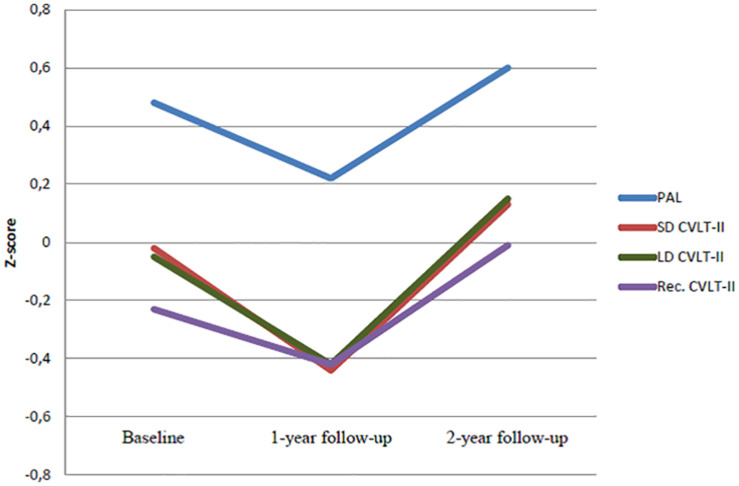
Repeated measures ANOVA of postoperative change in memory and verbal learning from baseline to 2-year follow-up (*n* = 48). PAL: Paired Associate Learning; CVLT: California Verbal Memory Test II; SD: short delay free recall; LD: long delay free recall; Rec: recognition(hits).

As shown in Table 2, mean test performance was within the normal range for all performance-based memory measures at baseline. Repeated measures ANOVAs (Table 2 and Figure 2) revealed a significant main effect of time for visual memory and verbal recall and recognition memory after surgery. Visual memory (PAL) [*F*(1.26, 60.52) = 9.38, *p* = 0.001, η^2^*_*p*_* = 0.16] showed a statistically significant reduction from baseline to 1-year follow-up (MD = 0.26, *p* = 0.043), and a subsequent significant improvement from 1- to 2-year follow-up (MD = −0.38, *p* = 0.002). There was no difference between the performance at baseline and 2-year follow-up (MD = −0.11, *p* = 0.056). A similar pattern of results was found for SD free recall (CVLT-II) [*F*(1.85, 87.08) = 10.30, *p* = 0.001, η^2^*_*p*_* = 0.18] and LD free recall (CVLT-II) [*F*(1.77, 83.50) = 12.25, *p* = 0.001, η^2^*_*p*_* = 0.19]. Both SD free recall (MD = 0.41, *p* = 0.016) and LD free recall (MD = 0.36, *p* = 0.009) worsened significantly from baseline to 1 year. The SD free recall (MD = −0.57, *p* = 0.001) and LD free recall performance (MD = −0.58, *p* = 0.001) significantly improved from 1- to 2-year follow-up. There was also a significant effect of time on recognition [*F*(1.9, 90.11) = 5.83, *p* = 0.004, η^2^*_*p*_* = 0.11], where the performance significantly improved from 1- to 2-year follow-up (MD = −0.42, *p* = 0.006). There was no significant main effect of time on verbal learning [*F*(1.78, 83.76) = 2.52, *p* = 0.078, η^2^*_*p*_* = 0.05]. As previous studies (Gunstad et al., 2011; Miller et al., 2013; Alosco et al., 2014a,b) have almost exclusively investigated patients undergoing RYGBP, *post-hoc* repeated measures ANOVAs were performed to investigate if the results were replicable for the subgroup of participants (54%, *n* = 26) that underwent RYGBP. The results for the RYGBP- group (data not shown) were indistinguishable to the results found for the sample as a whole.

**TABLE 2:** Repeated measures ANOVA for memory and verbal learning before and after surgery (*n* = 48).

	Baseline		1-year follow-up		2-year follow-up						
	*M* (*SD*)	Range	*M* (*SD*)	Range	*M* (*SD*)	Range	*df*	*F*	*p*	η^2^*_*p*_*	Sig. p.c.
Visual memory (PAL)	0.48 (0.38)	−0.47 to 0.98	0.22 (0.77)	−2.4 to 1.33	0.60 (0.35)	−0.27 to 0.98	1.26, 60.52	9.38	0.001	0.16	B-1, 1–2
SD free recall (CVLT-II)	−0.02 (0.94)	−1.5 to 2.0	−0.44 (1.06)	−2.5 to 2.0	0.13 (0.91)	−1.0 to 2.0	1.85, 87.08	10.30	0.001	0.18	B-1, 1–2
LD free recall (CVLT-II)	−0.05 (1.03)	−2.0 to 1.5	−0.42 (0.91)	−2.0 to 1.50	0.14 (0.92)	−1.5 to 1.5	1.77, 83.50	12.25	0.001	0.19	B-1, 1–2
Recognition (hits) (CVLT-II)	−0.24 (0.80)	−3.0 to 1.0	−0.42 (0.86)	−3.0 to 0.50	−0.01 (0.50)	−1.50 to 1.0	1.9, 90.11	5.83	0.004	0.11	1–2
Verbal learning (CVLT-II)	49 (10)	27–73	47 (10)	29–71	50 (10)	31–71	1.78, 83.76	2.52	0.078	0.05	
Everyday memory (EMQ-R)	12 (0.10)	0–44	13 (12)	0–46	12 (11)	0–38	1.4, 66.84	0.53	0.528	0.01	

**PAL, Paired Associate Learning (z-score); CVLT-II, California Verbal Memory Test II; SD, short delay (z-score); LD, long delay (z-score); EMQ-R, Everyday Memory Questionnaire Revised (total sum score); Verbal learning (T-score).**

### Self-Reported Everyday Memory (EMQ-R)

There was no main effect of time on the *presence* of self-reported everyday memory [*F*(1.4, 66.84) = 0.53, *p* = 0.528, η^2^*_*p*_* = 0.01] after surgery (Table 2). EMQ scores *averaged* over all items showed that participants overall reported experiencing everyday memory problems more than once a month but less than once a week (1) both before and after surgery; baseline mean (M) = 0.93, *SD* = 0.11; 1-year follow-up *M* = 1.08, *SD* = 0.14 and 2-year follow-up *M* = 0.96, *SD* = 0.12.

The mean score to each EMQ-R item (1–13) at each time point (baseline and 1- and 2-years follow-up) showed that before surgery the highest mean score was reported on item 5 *Finding that a word is “on the tip of your tongue”* (*M* = 1.48, *SD* = 1.3) and item 1 *Having to check whether you have done something that you should have done (M* = 1.31, *SD* = 1.3). At 1- and 2-year follow-up the highest mean scores was also reported on item 5 (1 year *M* = 1.78, *SD* = 1.3; 2 year *M* = 1.68, *SD* = 1.3) and item 1 (1 year *M* = 1.45, *SD* = 1.4; 2-year *M* = 1.20, *SD* = 1.3).

### Predictors of Change in Memory Performance After Surgery

The repeated measures ANOVAs revealed significant changes from baseline to 1-year follow-up in visual memory, SD free recall and LD free recall; hence, the subsequent regression analyses were only performed for these memory measures. Preliminary simple regression analyses showed that absolute weight change and comorbidity (CRP-level change and the presence of T2D, sleep apnea and hypertension) at 1-year follow-up did not predict change in memory performance at the predetermined level of significance (*p*-level = 0.15), and were therefore not included in further analyses.

## Discussion

The aim of this study was to investigate verbal, visual, and everyday memory at 1 and 2 years following bariatric surgery. The average level of performance-based memory was within the normal range both prior to and following surgery. Findings revealed that visual and verbal memory performance was significantly poorer at 1-year post-surgery with performance rebounding to baseline levels at 2-year follow-up. Verbal learning and self-reported memory problems showed no significant changes following surgery. Additionally, there was clear variability in memory test scores, with a small proportion of the participants showing impaired performance at both baseline and 1-year follow-up, but only marginally after 2 years.

Interestingly, these results are partly inconsistent with findings from a number of previous studies. Studies based upon Longitudinal Assessment of Bariatric Surgery (LABS), for example, have found moderate improvements for all verbal memory domains (SD free recall, LD free recall, recognition) at several follow-up points after surgery (12 weeks, 1-, 2-, and 3-years) (Gunstad et al., 2011; Miller et al., 2013; Alosco et al., 2014a,b). Further, these studies have found that the rate of patients with clinically significant memory impairment decreased after surgery (Gunstad et al., 2011; Miller et al., 2013; Alosco et al., 2014a,b). In contrast, the current study found that both visual and verbal postoperative memory performance at 1-year follow-up was significantly poorer compared to baseline. Further, the analyses of rates of impaired verbal memory performance partly diverged from prior findings. At baseline, impairment rates for the three selected CVLT-II measures were comparable to prior studies (Gunstad et al., 2011; Miller et al., 2013; Alosco et al., 2014b). Yet, in contrast to prior reports (Gunstad et al., 2011; Miller et al., 2013; Alosco et al., 2014b) 1-year follow-up rates in the current study remained at baseline levels. Our contradictory results are unlikely due to differences in sample composition. At baseline, our sample’s demographical characteristics, presence of medical comorbidity, and BMI were proportionate to most of the previous studies (Gunstad et al., 2011; Miller et al., 2013; Alosco et al., 2014a,b). In addition, the rate of patients showing memory impairment at baseline in this study seemed comparable to earlier findings (Gunstad et al., 2011; Miller et al., 2013; Alosco et al., 2014a,b). However, although LABS and the current study both assessed verbal memory, there were differences in test construction and format that may have affected test performance. For instance, the CVLT-II has been shown to be more demanding and consequently, more sensitive to subtle memory changes than the computerized list-learning task used in the LABS study (Lezak, 2012). In addition, CVLT-II performance is more dependent on executive function abilities (Hill et al., 2012). Hence, the inconsistency between studies may partly be due to differences in task demands. Another alternative that is specific for the CVLT-II, concerns the use of the alternate form at 1-year follow-up. In order to reduce the risk of practice effects (Benedict, 2005), which also follow at long test intervals (Alioto et al., 2017), the alternate form was used at 1-year follow-up. The alternate form has been criticized for not being equivalent to the standard form, and the test-retest reliability for the standard/alternate form has been reported as slightly lower than standard/standard form test-retest reliability (Woods et al., 2006). Hence, the poorer verbal memory performance at 1 year might partly reflect reduced test-retest reliability. However, findings for *visual* memory followed the same pattern as verbal memory, with poorer performance 1 year following surgery. As such, the use of the alternate CVLT-II form at 1 year may not offer a full explanation of discrepant study findings. Rather, the results might reveal a temporarily reduction in both visual and verbal memory performance 1 year after surgery. However, it is important to note, that although the effect sizes for the main effect (time) was large, the mean test scores were within normal variation at all follow-up points. As such, the overall clinical significance of these findings is uncertain.

Another main finding involves the lack of change in subjectively reported everyday memory problems after surgery. Overall, the participants reported experiencing memory problems more than once a month, but less than once a week, both before and after surgery. Since the EMQ-R is without cut-off scores to indicate clinical pathology, it is difficult to determine the clinical implication of this finding. Compared to results from one prior study using the EMQ-R (Royle and Lincoln, 2008), the participants in the current study reported having memory problems more often than normal controls, and at the same level as patients with multiple sclerosis. One prior study of bariatric surgery candidates (Garcia et al., 2013) found that subjectively experienced cognitive problems (3 generic items) did not correlate with test performance. The authors suggested that patients had limited insight into their level of cognitive functioning. A similar conclusion cannot be made based on the analyses performed in the current study. However, the EMQ-R results did not seem to reflect the patterns of results seen for the performance-based memory measures.

It is unclear why poorer performance in both verbal and visual memory occurred at 1-year follow-up, with a return to baseline levels at 2 years. Our study examined whether absolute weight loss, change in CRP-level, or the presence of comorbid disease at 1-year follow-up predicted change in postoperative memory performance. Consistent with most prior studies, none of these variables, however, significantly predicted postoperative memory (Gunstad et al., 2011; Miller et al., 2013; Alosco et al., 2014a,b). The results also showed that the 1-year follow-up rates of memory impairment were similar to baseline levels. The parallel improvement seen in comorbid disease at 1 year-follow up, corroborate that the changed memory performance at 1 year follow-up were unrelated to changes in comorbid disease. Other factors found to predict postoperative cognitive functioning (Spitznagel et al., 2015), such as improved glucose regulation (Galioto et al., 2015) and improvements in leptin and ghrelin levels (Alosco et al., 2015), were not assessed in the current study. Rejecting the possible influence of reduced standard/alternate test-retest reliability for the CVLT-II, one may speculate if the poorer visual and verbal memory performance at 1 year follow-up reflected transient effects of the surgical procedure, which also could influence memory functioning the first year after surgery, for instance nutritional deficiencies (Cornejo-Pareja et al., 2019), systemic stress response (Prete et al., 2018), or anesthesia effects (Wu et al., 2019). Nevertheless, these potential factors were presumably also present across all previous studies, and therefore, provide insufficient clarification to the finding of poorer postoperative memory at 1-year follow-up.

The main strengths of this study involved the use of performance-based assessments of both visual and verbal memory, and the use of a reliable and valid self-report measure of everyday memory problems. In addition, this study had a 1- and 2-years follow-up period. The present study also had some limitations to note. The single site data collection, convenience sampling and small sample size has, most likely, led to selection bias and reduced statistical power, weakening the generalizability of the findings. Also, potential confounding variables known to affect cognitive test performance (e.g., depression and use of medication) were not accounted for in the statistical analyses. However, the frequency of self-reported medication use was considered too low for inclusion in the statistical models. Lastly, the present work lacks a control group, which should be included in further studies to provide more solid conclusions regarding the specific effects of undergoing bariatric surgery.

## Conclusion

In conclusion, main findings were that visual and verbal memory performance was poorer 1 year after surgery before returning to baseline levels at 2 years, and that verbal memory impairment rates were unchanged at 1-year follow-up. In the context of the above-mentioned study limitations, results may indicate that postoperative memory improvements following bariatric surgery are not universal. Hence, treatment providers should also be alert of the possibility of patients having poorer memory 1 year following surgery, and that a proportion of their patients may experience clinically significant verbal memory problems both before and after surgery. Ideally, these results require replication in a study with a larger sample representative of all surgical procedures currently used in clinical practice. Pending generalizability of these findings to the bariatric surgery population-at-large, it would be relevant to establish whether memory changes observed in this study adversely affect patients’ eating behavior and their ability to adhere to postoperative treatment guidelines.

## Data Availability Statement

All datasets generated for this study are included in the article/Supplementary Material, further inquiries can be directed to the corresponding author.

## Ethics Statement

The studies involving human participants were reviewed and approved by the Regional Committee for Medical and Health Research Ethics of South-Eastern Norway. The patients/participants provided their written informed consent to participate in this study.

## Author Contributions

CD and IK conceived the original idea and designed the study. GW-S and IE collected the data. GW-S performed the analyses reported in the current study and wrote the manuscript. CB contributed to the data analysis. All authors contributed to the interpretation of the results and approved the final version of the manuscript.

## Conflict of Interest

The authors declare that the research was conducted in the absence of any commercial or financial relationships that could be construed as a potential conflict of interest.
