# Supplementary material for: Visual, Verbal and Everyday Memory 2 Years After Bariatric Surgery: Poorer Memory Performance at 1-Year Follow-Up
Source: Front Psychol. 2021 Jan 8;11:607834. doi: 10.3389/fpsyg.2020.607834 (PMC7820680; doi:10.3389/fpsyg.2020.607834)
Supplement: Supplementary file 1 [file Table_1.docx]

**Supplementary Table. Internal reliability and factor loadings of the Everyday Memory Questionnaire-Revised (EMQ-R).**

Table 1. Rotated factor loadings of the EMQ-R items.

| Item | Description | Component |  |
| --- | --- | --- | --- |
|  |  | 1 | 2 |
|  | Label | Retrieval | Attention |
|  | Cronbach`s α | .88 | .80 |
|  | Percentage of variance | 49.85% | 10.42% |
| 1 | Having to check whether you have done something that you should have done. | .86 |  |
| 3. | Forgetting that you were told something yesterday or a few days ago and maybe having to be reminded about it. | .81 |  |
| 5. | Finding that a word is ‘on the tip of your tongue’. You know what it is but cannot quite find it. | .79 |  |
| 6. | Completely forgetting to do things you said you would do, and things you planned to do. | .74 |  |
| 2. | Forgetting when it was that something happened; for example, whether it was yesterday or last week. | .70 |  |
| 7. | Forgetting important details of what you did or what happened to you the day before. | .68 |  |
| 10. | Forgetting to tell somebody something important, perhaps forgetting to pass on a message or remind someone of something. | .51 |  |
| 11. | Getting the details of what someone has told you mixed up and confused. | .44 | .45 |
| 12. | Forgetting where things are normally kept or looking for them in the wrong place. |  | .87 |
| 13. | Repeating to someone what you have just told them or asking someone the same question twice. |  | .82 |
| 8. | When talking to someone, forgetting what you have just said. Maybe saying ‘what was I talking about? |  | .68 |
| 4. | Starting to read something (a book or an article in a newspaper, or a magazine) without realizing you have already read it before. |  | .60 |
| 9. | When reading a newspaper or magazine, being unable to follow the thread of a story; losing track of what it is about. |  | .57 |

Note. EMQ-R: Everyday Memory Questionnaire-Revised. Data from all participants (n=80) at baseline. Additional information available upon request.
